# Systematic Influence of Circulating Bilirubin Levels on Osteoporosis

**DOI:** 10.3389/fendo.2021.719920

**Published:** 2021-08-26

**Authors:** Jinqiu Zhao, Muzi Zhang, Zhengxue Quan, Liang Deng, Yongguo Li, Bin He

**Affiliations:** ^1^Department of Infectious Diseases, The First Affiliated Hospital of Chongqing Medical University, Chongqing, China; ^2^Department of Orthopedics, The First Affiliated Hospital of Chongqing Medical University, Chongqing, China; ^3^Department of Gastroenterology, The First Affiliated Hospital of Chongqing Medical University, Chongqing, China

**Keywords:** osteoporosis, circulating bilirubin levels, BMD, fracture, Mendelian randomization study

## Abstract

Observational studies report some association between circulating bilirubin levels and osteoporosis, but it is unknown if this association is causal or confounded. In this two-sample Mendelian randomization (MR) study, we included a large genome-wide association study (GWAS) associated with total bilirubin levels among 317,639 people, a large meta-analysis to identify genetic variants associated with bone mineral density (BMD) estimated by heel quantitative ultrasound (eBMD) among 426,824 individuals and fracture among 1.2 million individuals. The results revealed that circulating bilirubin levels had no causal influence on eBMD (beta-estimate: 0.004, 95% confidence interval [CI]: -0.019 to 0.028, SE:0.012, P-value=0.705) or the risk of fracture (beta-estimate: -0.009, 95% CI: -0.035 to 0.017, SE:0.013, P-value=0.488), which were both confirmed by multiple sensitivity analyses. Our results confirm that circulating bilirubin levels have no causal role in eBMD or the incidence of fracture, indicating that circulating bilirubin levels is unlikely to be a causal risk factor for osteoporosis or fracture.

## Introduction

As one common and aging-related disease, osteoporosis is featured by decreased bone mineral density (BMD) and increased risk of fracture (1–4). The treatment of osteoporosis remains a big challenge and public health problem in the world (5–7). Osteoporosis is a common complication of liver diseases such as chronic cholestasis and primary biliary cholangitis (8, 9). Bone loss is caused by deficient osteoblast activity and increased bone resorption (10). High concentrations of circulating bilirubin levels were documented to result in the abnormal osteoblast function and serum bilirubin had detrimental effects on bone-forming cells (11). Emerging evidence suggested that total bilirubin levels participated in multiple biological activities such as immunomodulatory processes (12, 13).

The association between bilirubin levels and osteoporosis has not been well established, and several studies have reported the conflicting results (8, 14–16). For instance, one observational study included 918 postmenopausal individuals without potential liver diseases, and revealed that total bilirubin level was independently associated with BMD [beta-estimate=0.41, 95% CI (0.35–0.47), P<0.001 for lumbar spine BMD and beta-estimate=0.44, 95% CI (0.36–0.48), P<0.001 for femur neck BMD] (16). However, these observational studies may be subject to confounding factors and reverse causality.

Genome-wide association studies (GWASs) demonstrate that osteoporosis is one highly polygenic trait (17–19). Mendelian randomization (MR) study is effective and powerful to establish the causal relationship between exposure phenotype and exposure phenotype through using the GWAS summary statistics (20–22). These genetic variants are randomly allocated before birth and fixed at conception, and benefit to prevent reverse causation and potential confounding factors (23, 24).

The two-sample MR analysis has emerged as an important approach to greatly increase the scope and statistical power of MR based on the published summary data from GWASs (22, 25, 26). In this study, we use single nucleotide polymorphisms (SNPs) strongly associated with circulating bilirubin levels as the instrumental variables. To our knowledge, this is the first two-sample MR study to explore the causal effect of total bilirubin levels on BMD and fracture.

## Methods

### Genetic Instrument for Circulating Bilirubin Levels

A large-scale GWAS associated with circulating bilirubin levels included 317,639 individuals of European ancestry (27). Total bilirubin levels in serum was measured using a colorimetric assay (2,4-dicholorani-line reaction). Each SNP was tested after adjusting for age, sex, recruitment center, indicators of socioeconomic status, the top 40 principal components for population stratification, and potential technical confounders (i.e. fasting time, sample dilution factor, blood and urine sampling time) (27).

Initially, 115 SNPs with genome-wide significance (P<5×10^−8^) were found to have robust association with total bilirubin levels (**Supplementary Table 1**). Then, SNPs were ideally expected to not be in linkage disequilibrium (LD), because SNPs in strong LD may produce some bias. We measured LD between selected SNPs using European samples from the 1000 Genomes project. 27 SNPs were excluded due to high LD (r^2^≥0.001). Finally, 88 SNPs associated with circulating bilirubin levels were used as instrumental variables (**Supplementary Table 2**). If SNPs were unavailable in the outcome dataset, the proxy SNPs in linkage disequilibrium (LD, r^2^>0.9) were used as the instrumental variables. Thus, rs72831372 was used as a proxy for rs7185774 among eBMD, but no proxy SNPs were used for rs11601507 or rs157595 among eBMD (**Supplementary Table 2**).

### Data Sources of eBMD and Fracture

The large-scale GWAS summary data was calculated among 426,824 people of European decent for bone mineral density (BMD) and up to 1.2 million individuals of European decent for fracture. BMD was estimated by heel quantitative ultrasound (eBMD, [g/cm^2^]), which was derived as a linear combination of speed of sound (SOS) and bone ultrasound attenuation (BUA) (i.e. eBMD = 0.002592 * (BUA + SOS) − 3.687). Fracture cases were defined as any fracture apart from the fracture of skull, face, hands, feet, pathological fractures due to malignancy, atypical femoral fractures, periprosthetic and healed fracture. These GWAS summary data were analyzed after adjusting for age, sex and genotyping (1).

### Statistical Analyses

To determine MR estimates of total bilirubin levels on eBMD and fracture, we conducted the inverse variance weighted (IVW) meta-analysis of the Wald ratio. The weighted median and MR-Egger regression methods were also applied to estimate the effects. We evaluated the directional pleiotropy based on the intercept obtained from MR-Egger analysis (28). MR pleiotropy residual sum and outlier test (MR-PRESSO) was also used to assess the presence of pleiotropy and the effect estimates were recalculated after outlying SNPs were excluded (29).

The ethical approval for each study included in this investigation can be found in the original publications (including informed consent from each participant). The differences with P<0.05 were considered statistically significant. All of these analyses were conducted in R V.4.0.4 by using the R packages of ‘MendelianRandomization’ (30), ‘TwoSampleMR’ (31) and ‘MR-PRESSO’ (32).

## Results

### Causal Effect of Circulating Bilirubin Levels on eBMD

**Figure 1:**
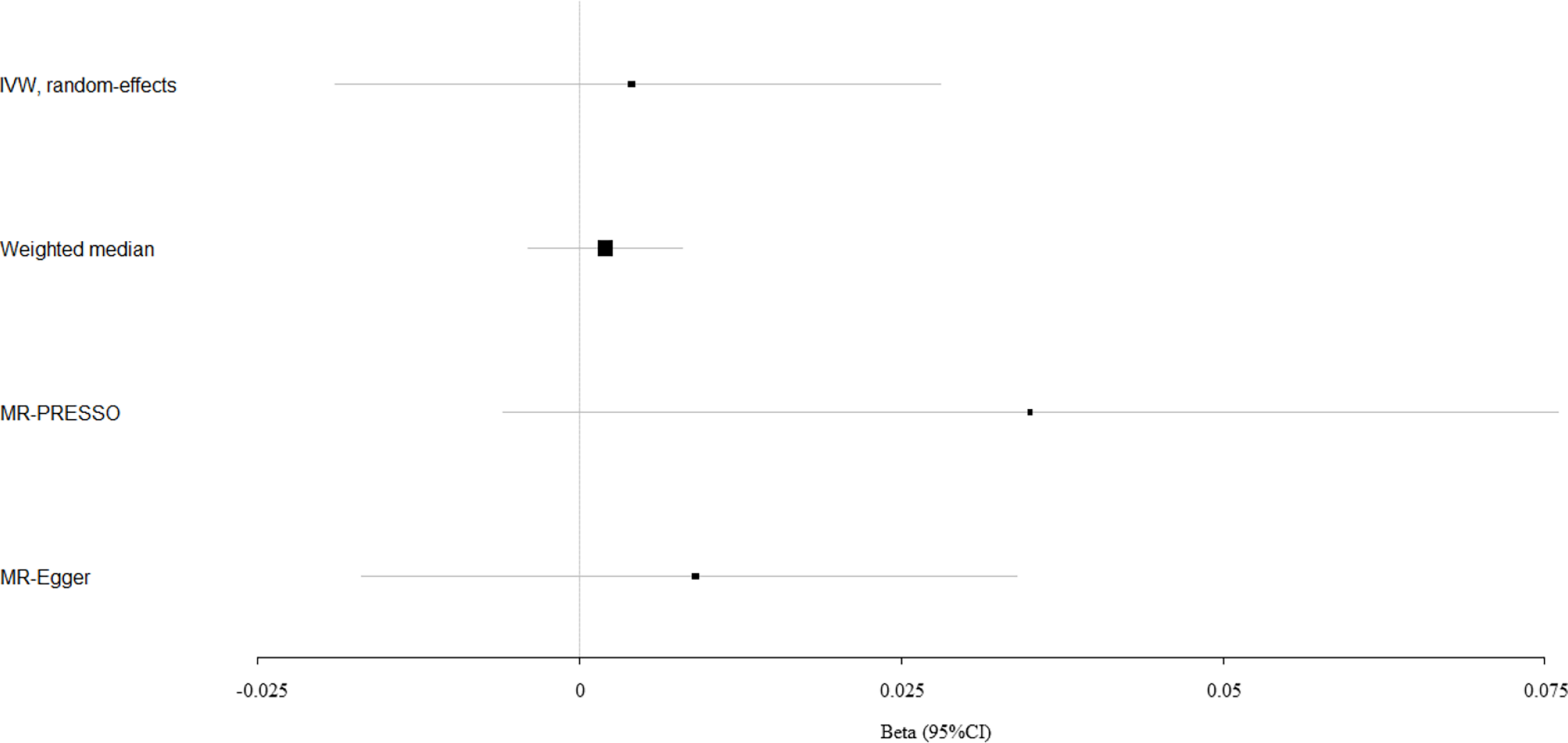
Mendelian randomization analysis for the association between circulating bilirubin levels and eBMD.

We evaluated the causal effect of circulating bilirubin levels on eBMD in the MR analysis (**Table 1**). Circulating bilirubin levels demonstrated no obvious MR association with eBMD according to IVW analysis (beta-estimate: 0.004, 95% CI: -0.019 to 0.028, SE:0.012, P-value=0.705) or weighted-median analysis (beta-estimate: 0.002, 95% CI: -0.004 to 0.008, SE:0.003, P-value=0.511), which was also confirmed by MR-Egger analysis (beta-estimate: 0.009, 95% CI: -0.017 to 0.034, SE:0.013, P-value=0.500, **Table 1** and **Figure 1**).

**Table 1:** Mendelian randomization estimates of bilirubin levels on osteoporosis.

Variables	IVW							Weighted median				MR-Egger							
	Estimate	SE	95% CI	P-value	Q value	I^2^	Heterogeneity P value	Estimate	SE	95% CI	P-value	Estimate	SE	95% CI	P-value	Intercept	SE	95% CI	Pleiotropy P value
eBMD	0.004	0.012	-0.019,0.028	0.705	1360.315	93.80%	0.000	0.002	0.003	-0.004,0.008	0.511	0.009	0.013	-0.017,0.034	0.500	-0.001	0.001	-0.003,0.001	0.406
Fracture	-0.009	0.013	-0.035,0.017	0.488	136.326	70.40%	0.001	-0.009	0.011	-0.031,0.013	0.436	-0.007	0.014	-0.035,0.021	0.618	0.000	0.001	-0.003,0.002	0.732

### Causal Effect of Circulating Bilirubin Levels on Fracture

**Figure 2:**
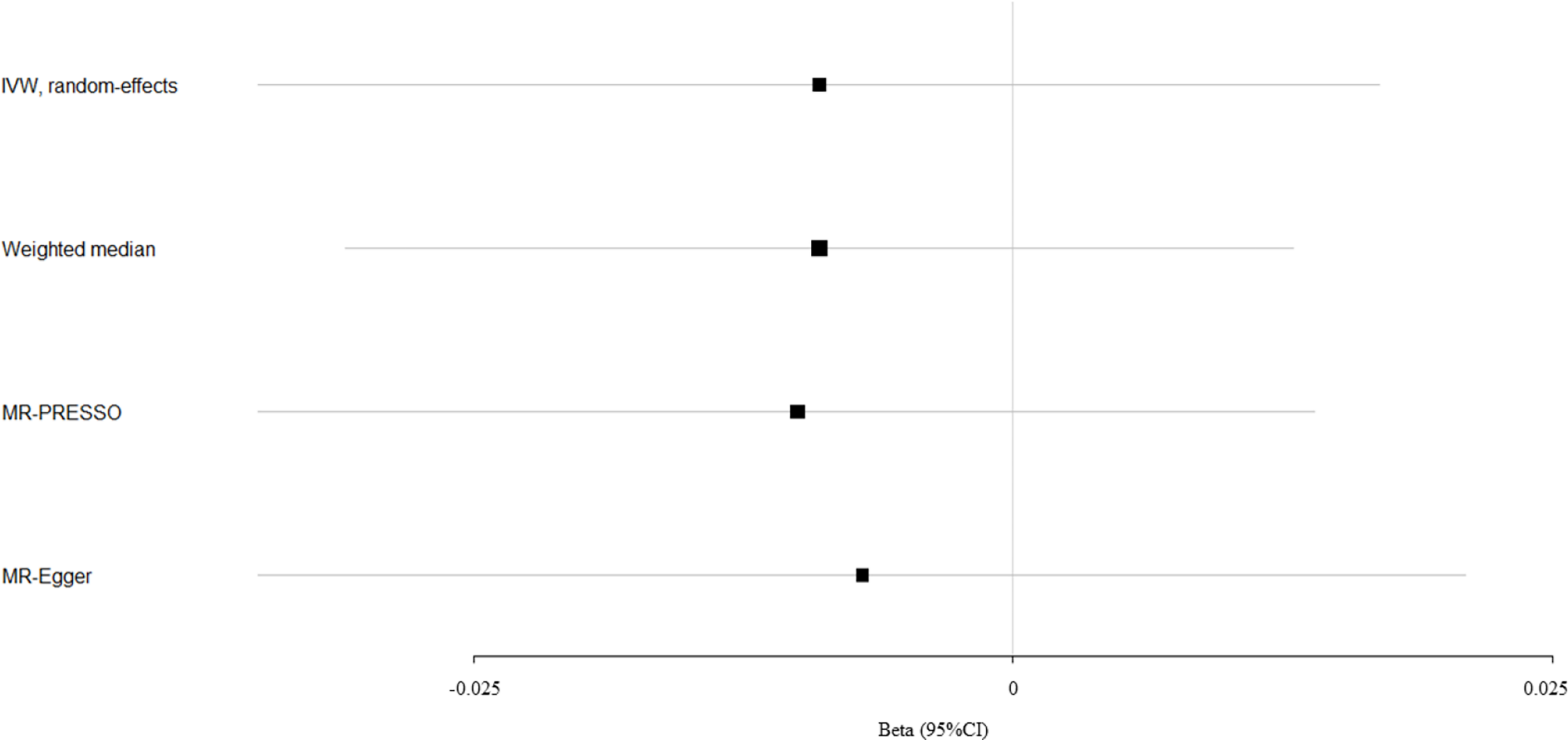
Mendelian randomization analysis for the association between circulating bilirubin levels and fracture.

Circulating bilirubin levels showed no causal effect on the risk of fracture according to the IVW analysis (beta-estimate: -0.009, 95% CI: -0.035 to 0.017, SE:0.013, P-value=0.488), weighted-median analysis (beta-estimate: -0.009, 95% CI: -0.031 to 0.013, SE:0.011, P-value=0.436) or MR-Egger analysis (beta-estimate: -0.007, 95% CI: -0.035 to 0.021, SE:0.014, P-value=0.618, **Table 1**). MR association between circulating bilirubin levels and fracture was presented in **Figure 2**.

### Evaluation of Assumptions and Sensitivity Analyses

There was little evidence of directional pleiotropy for all models (MR-Egger intercept P-values >0.05) (**Table 1**). Significant heterogeneity remained for eBMD and fracture based on Cochran’s Q (Heterogeneity P-heterogeneity<0.05), and thus MR-PRESSO test was conducted to identify 27 outliers (rs2375279, rs17513135, rs556107, rs4671605, rs6431625, rs2267846, rs1482852, rs61791066, rs151450, rs1126673, rs6869704, rs12515233, rs853684, rs12210538, rs58699591, rs3118753, rs2519093, rs34755157, rs174554, rs76895963, rs4149056, rs4760682, rs61984409, rs17184256, rs4575545, rs7222046, rs4820091) for eBMD and one outlier (rs174554) for fracture among the 88 SNP instrumental variables.

After excluding these outlying SNP variants, circulating bilirubin levels showed no MR association with eBMD (beta-estimate: 0.035, 95% CI: -0.006 to 0.076, SE:0.021, P-value=0.094, **Table 2** and **Figure 1**) or fracture (beta-estimate: -0.010, 95% CI: -0.035 to 0.014, SE:0.012, P-value=0.402, **Table 2** and **Figure 2**).

**Table 2:** Mendelian randomization estimates between bilirubin levels and osteopross after excluding outliers detected by PRESSO.

Variables	Estimate	SE	95% CI	P-value
eBMD excluding 27 outliers (rs2375279, rs17513135, rs556107, rs4671605, rs6431625, rs2267846, rs1482852, rs61791066, rs151450, rs1126673, rs6869704, rs12515233, rs853684, rs12210538, rs58699591, rs3118753, rs2519093, rs34755157, rs174554, rs76895963, rs4149056, rs4760682, rs61984409, rs17184256, rs4575545, rs7222046, rs4820091)	0.035	0.021	-0.006,0.076	0.094
Fracture excluding one outlier (rs174554)	-0.01	0.012	-0.035,0.014	0.402

## Discussion

Overall, our large multi-instrument approaches found no causal effect of circulating bilirubin levels on eBMD or the risk of fracture. This two-sample MR estimates had great robustness to support no MR association between circulating bilirubin levels and osteoporosis based on the results of various MR methods and sensitivity analyses. These indicated that circulating bilirubin levels is not the causal risk factor for osteoporosis or fracture.

Circulating bilirubin level is one robust indicator of hepatic dysfunction, and *in vitro* study reveals that bilirubin from jaundiced patients can inhibit the proliferative capacity of osteoblast (11, 33, 34). Total bilirubin has a detrimental effect on cell viability, cell differentiation and mineralization of primary human osteoblasts in a dose-dependent manner (11). In addition, total bilirubin is an end metabolic product of heme degradation and servers as a potent antioxidant to inhibit the oxidization of lipids and lipoprotein (especially low density lipoprotein) and eliminate radical oxygen species in the various forms including free, albumin-bound, unconjugated and conjugated forms (16, 35). Osteoporosis caused by oxidative stress may be also associated with endothelial dysfunction and decreased blood flow for bone tissue (36, 37). These provide strong theoretical support for the association between circulating bilirubin levels and osteoporosis.

Several epidemiological studies have demonstrated the significant inverse association between total bilirubin levels and BMD in patients with underlying liver diseases, but other studies reported conflicting association between them (8, 14–16). This inconsistent association may be caused by the methodological limitations (i.e. confounding, reverse causation and measurement error) of traditional observational study and small patient sample (38). Because total bilirubin level is a highly informative indicator of hepatic dysfunction, it is infeasible to identify whether bilirubin level is the direct cause of osteoporosis (16, 33, 39). It remains uncertain whether circulating bilirubin level has the casual effect on osteoporosis or fracture.

Randomized controlled trial (RCT) is the gold standard in causal inference, but it is not feasible to explore the association between total bilirubin levels and osteoporosis due to the unethical approaches of raising bilirubin levels. The MR study is widely used to evaluate causal inferences between risk factors and disease outcomes with the features of preventing confounding and reverse causation (40). To date, our work is the first two-sample MR study to explore the causal effect of circulating bilirubin levels on eBMD and fracture.

Our study included the large GWAS for total bilirubin levels among 317,639 individuals and the large meta-analysis to identify genetic variants associated with BMD among 426,824 individuals and fracture among 1.2 million individuals. This two-sample MR study confirmed no casual role of circulating bilirubin levels in eBMD or the risk of fracture based on the multiple MR methods and sensitivity analyses.

There are several important strengths in this study. This is the first two-sample MR study to investigate the causal effect of circulating bilirubin levels on BMD and fracture. This study design can prevent some limitations (e.g. reverse causation and potential confounding factors) of conventional observational studies. Our study include large sample sizes of GWAS summary data associated with circulating bilirubin levels, BMD and fracture. The intercepts for the MR-Egger analyses suggest that all causal associations are not affected by directional pleiotropy. We also conduct multiple sensitivity analyses to test the influence of pleiotropy on our causal estimates, and our results are consistent and robust according to various MR tests and sensitivity analyses.

Several limitations should be taken into consideration. Firstly, all the included participants are of European decent, and more studies are needed to explore whether our findings are generalizable to other populations. Secondly, it is not feasible to perform the MR analysis based on different age stratums because of the limitation of GWAS summary statistics. Thirdly, significant heterogeneity remains for the MR association between bilirubin levels and BMD/fracture, which may be caused by different patient populations and unknown confounding factors.

## Conclusion

This two-sample MR provides strong evidence to confirm that circulating bilirubin levels is unlikely to be a causal risk factor of osteoporosis or fracture.

## Data Availability Statement

The original contributions presented in the study are included in the article/**Supplementary Material**. Further inquiries can be directed to the corresponding authors.

## Author Contributions

JZ, MZ, BH, and ZQ conducted Study Design, Data Collection, and Statistical Analysis. BH, MZ, LD, YL, and JZ conducted Data Interpretation, Manuscript Preparation, and Literature Search. LD, BH, and JZ conducted Funds Collection. All authors contributed to the article and approved the submitted version.

## Funding

This study was funded by Chongqing Yuzhong Nature Science Foundation of China (Grant No. 2018114), National Natural Science Foundation of China (82070626) and Natural Science Foundation of Chongqing (cstc2019jcyj-msxmX0836).

## Conflict of Interest

The authors declare that the research was conducted in the absence of any commercial or financial relationships that could be construed as a potential conflict of interest.

## Publisher’s Note

All claims expressed in this article are solely those of the authors and do not necessarily represent those of their affiliated organizations, or those of the publisher, the editors and the reviewers. Any product that may be evaluated in this article, or claim that may be made by its manufacturer, is not guaranteed or endorsed by the publisher.
